# Urinary Tract Infections Caused by Extended-Spectrum Beta-Lactamase-Producing and Carbapenem-Resistant Enterobacterales in Saudi Arabia: Impact of Catheterization

**DOI:** 10.3390/medicina61111907

**Published:** 2025-10-24

**Authors:** Asma Ali Sawan, Nada S. Alghamdi, Shahad A. Alzahrani, Muzn S. Alharbi, Nora Alabdulkareem, Dana Ahmed Alnufaily, Sajidah Jaffar Alalwan, Tajammal Mustafa, Maher Alqurashi, Ayman A. El-Badry

**Affiliations:** 1 Department of Microbiology, College of Medicine, Imam Abdulrahman bin Faisal University, Dammam 31441, Saudi Arabia; nsaghamdi@iau.edu.sa (N.S.A.); msalqurashi@iau.edu.sa (M.A.); or aelbadry@kasralainy.edu.eg (A.A.E.-B.); 2 College of Medicine, Imam Abdulrahman Bin Faisal University, Dammam 31441, Saudi Arabia; mozn.alharbi@gmail.com (M.S.A.); noraalabdulkareem@gmail.com (N.A.); dana.alnufaily@gmail.com (D.A.A.); sajidah2011@gmail.com (S.J.A.); 3 Family and Community Medicine Department, College of Medicine, Imam Abdulrahman Bin Faisal University, Dammam 31441, Saudi Arabia; tmostafa@iau.edu.sa

**Keywords:** urinary tract infection, ESBL, CRE, Enterobacterales, catheterization, antimicrobial resistance, catheter-associated urinary tract infection (CAUTI)

## Abstract

*Background and Objectives*: Catheter-associated urinary tract infections (CAUTIs) caused by extended-spectrum beta-lactamase (ESBL)-producing and carbapenem-resistant Enterobacterales (CRE) significantly contribute to global rates of UTI. This study aimed to compare the prevalence and trends of ESBL-producing Enterobacterales and CRE in patients with CAUTIs and non-CAUTIs. *Materials and Methods*: A retrospective review of 4262 UTI-positive urine cultures was conducted at King Fahad Hospital of the University, Al Khobar, Saudi Arabia (January 2022–November 2023). Demographic, clinical, and microbiological data were obtained from hospital records. Antimicrobial susceptibility was tested using the Vitek^®^ System; ESBL and CRE were identified using Ezy MIC™ strips and Xpert^®^ Carba-R assay, respectively. *Results*: ESBL-producing Enterobacterales accounted for 11.3% of cases; CRE comprised 1.8%. ESBL was significantly more prevalent in non-catheterized patients and those in emergency care. CRE was significantly associated with catheterized patients and inpatient settings. *Escherichia coli* and *Klebsiella pneumoniae* were the predominant ESBL-producing and CRE isolates, respectively. *bla-OXA-48* was the most frequently detected carbapenemase gene (66.7%). ESBL was prevalent in younger, non-catheterized females, suggesting increasing community transmission. Conversely, CRE were primarily observed in older, catheterized inpatients, emphasizing the role of invasive devices in resistance spread. *Conclusions*: These findings highlight the importance of targeted infection control and early catheter removal to mitigate resistance trends.

## 1. Introduction

Urinary tract infections (UTIs) pose a significant global public health concern, with an estimated 404.6 million incident cases and 236,786 deaths in 2019 [1]. In Saudi Arabia, UTIs represent 10% of all infections and are the second most common cause of emergency department admissions [2]. The global burden of UTIs has increased due to rising treatment failure driven by multidrug-resistant uropathogens.

UTIs are typically classified into catheter-associated urinary tract infections (CAUTIs) and non-catheter-associated UTIs (non-CAUTIs). CAUTIs arise when an indwelling catheter provides direct access for pathogens into the urinary tract, accounting for 70–80% of hospital-acquired UTI (HAUTI) [3]. CAUTI rates are 1.3 per 1000 catheter days in high-income countries and 3.16 per 1000 catheter days in low- and middle-income countries [4].

Most CAUTIs are caused by Enterobacterales, particularly *Escherichia coli*, the most common uropathogen [5]. Enterobacterales are facultatively anaerobic Gram-negative bacteria that include clinically significant pathogens such as *Escherichia coli*, *Klebsiella pneumoniae*, *Enterobacter cloacae*, *Proteus mirabilis*, *Citrobacter freundii*, and *Morganella morganii*, *among others* [6]. Colonization occurs via biofilm formation on catheter surfaces, shielding bacteria from the host immune response and antibiotic activity [7]. Consequently, urine stasis and biofilm formation make catheterized patients highly susceptible to drug-resistant strains [8]. Colonizing organisms are often ESBL-producing Enterobacterales or carbapenem-resistant Enterobacterales (CRE). ESBL-producing bacteria are resistant to all beta-lactam antibiotics (e.g., penicillins and cephalosporins), while CRE strains are resistant to carbapenems, thereby significantly limiting treatment options [9].

The growing burden of resistance is becoming increasingly evident. For instance, 33.49% of *E. coli* isolates from UTI cases in Riyadh were ESBL-producing [10]. Moreover, ESBL-producing microbes are more frequently isolated in catheter-associated UTIs (65.33%) than in non-catheter-associated cases (47.66%) [11], underscoring the role of catheterization in fostering resistant infections. The rising incidence of ESBL and CRE in UTIs leads to treatment failure, prolonged hospital stays, and elevated healthcare costs [10,12]. The inability to identify effective antimicrobials complicates empirical therapy and necessitates the use of last-resort agents, which often have serious adverse effects [12]. The presence of ESBL and CRE traits in Enterobacterales substantially reduces the efficacy of conventional therapies, resulting in infection recurrence, sepsis, and increased mortality. Additionally, catheterized individuals face a heightened risk of sepsis, which complicates clinical management and further increases the likelihood of poor outcomes [13,14].

The main aim of this study was to analyze the prevalence and trends of ESBL-producing Enterobacterales and CRE in UTIs in urine samples among catheterized (CAUTI) and non-catheterized (non-CAUTI) patients. Specifically, we sought to (1) determine the prevalence of ESBL-producing Enterobacterales and CRE among patients with Enterobacterales cultures, with a focus on differences between catheterized and non-catheterized individuals, (2) identify the predominant bacterial species associated with ESBL-producing Enterobacterales and CRE, as well as the distribution of resistance genes involved, and (3) explore patient characteristics (demographic, clinical, laboratory, and catheter use) as potential risk factors for the occurrence of ESBL-producing Enterobacterales and CRE in the study population.

## 2. Materials and Methods

### 2.1. Subjects

This is a retrospective study conducted at King Fahad Hospital of the University in Al Khobar, Saudi Arabia, between January 2022 and November 2023.

Inclusion criteria encompassed all patients with positive urine cultures from January 2022 to November 2023. There were no exclusion criteria. A total of 4262 urine samples were collected using a non-probability consecutive sampling method from the hospital’s electronic medical records. Patient demographic and clinical data were also extracted, including sex, age, and care setting.

The research was conducted following ethical approval from the Institutional Review Board (IRB) of Imam Abdulrahman bin Faisal University under approval number IRB-UGS-2024-01-730. All procedures adhered to applicable ethical standards. Data collected were kept confidential and used solely for the purpose of this study. The Institutional Review Board of Imam Abdulrahman Bin Faisal University, Saudi Arabia, granted a waiver of informed consent due to the retrospective study design.

### 2.2. Variables

In this study, the primary independent variable is the patient’s catheterization status, which was stratified to evaluate its impact on outcomes. This variable was divided into two subgroups: those with indwelling catheters and those without catheters. The type of urine specimen collected—such as random, midstream, suprapubic bladder, catheterized, or urine bag—was also considered an independent variable, allowing further stratification in the analysis. Additional independent variables included patient demographics such as sex, age, and nationality, as well as clinical characteristics related to care setting. Care settings were categorized as outpatient (primarily clinics), inpatient (including wards and ICUs), and the Emergency Department.

The dependent variables include the presence of Enterobacterales in urine cultures, molecular confirmation of ESBL and CRE, and antimicrobial susceptibility testing (AST) results. Enterobacterales isolates resistant to at least one third-generation cephalosporin underwent manual ESBL testing using ESBL Ezy MIC™ Strips (Himedia Labs, Thane, India). Isolates resistant to at least one carbapenem underwent molecular testing with the Xpert^®^ Carba-R for rapid detection and differentiation of *bla-KPC*, *bla-NDM*, *bla-VIM*, *bla-IMP*, and *bla-OXA-48* gene sequences from pure colonies.

Uniform microbiological and molecular testing procedures were applied to all urine specimens. To prevent case duplication, patients were considered distinct if different isolates were recovered or if the same isolate was detected at least 14 days apart.

### 2.3. Procedures

First, all urine cultures from the study period were extracted from King Fahd University Hospital Electronic Medical Records (EMR; TrakCare Electronic Medical Record System). Subsequently, eligible patients were identified through systematic searches in the EMR and the hospital’s laboratory databases based on positive Enterobacterales urine cultures. Relevant patient data, including demographic and clinical information, were then collected and documented using Microsoft Excel (Version 16.64).

Antibiotic susceptibility testing was routinely conducted on all isolates according to the protocol of the local laboratory. The Vitek^®^ automated system (BioMérieux, Marcy-l’Étoile, France) was used to determine the minimum inhibitory concentration (MIC) of antibiotics against the isolated bacteria. AST results were displayed and interpreted as sensitive (S), intermediate (I), or resistant (R), in accordance with Clinical and Laboratory Standards Institute guidelines [15]. Isolates resistant to at least one third-generation cephalosporin underwent confirmatory ESBL testing using ESBL Ezy MIC™ Strips (Himedia Labs, Thane, India) as part of routine laboratory procedure.

Additionally, isolates resistant to at least one carbapenem were subjected to molecular testing using the Xpert^®^ Carba-R assay (Cepheid, Sunnyvale, CA, USA) for rapid detection and differentiation of the *bla-KPC*, *bla-NDM*, *bla-VIM*, *bla-IMP*, and *bla-OXA-48* gene families.

### 2.4. Statistical Analysis

The data were presented as numbers and percentages for all categorical variables. ESBL, CRE, and specimen type were compared based on the demographic and clinical characteristics of the patients using Fisher’s Exact test and Chi-square test. Statistical significance was set at *p* < 0.05. All analyses were performed using SPSS version 26 (IBM Corp., Armonk, NY, USA).

## 3. Results

### 3.1. Overall Prevalence of ESBL and CRE in Enterobacterales Isolates: Comparison Between Catheterized and Non-Catheterized Patients

Analysis of the results showed that ESBL-producing organisms had higher overall detection rates than CRE, with differing prevalence trends between the two groups. Specifically, ESBL-producing bacteria were primarily associated with emergency settings and non-catheterized samples, whereas CRE were more frequently identified in inpatient settings and catheterized samples.

Supplementary Table S1 shows that patients over 70 years old comprised 18.7% of the cases, females represented 69% of the study population, and Saudi nationals accounted for 83.1%. The highest proportion of infections occurred in outpatient emergency settings (55.8%), followed by outpatient clinics (21.2%) and inpatient medical wards (12%).

**Figure 1:**
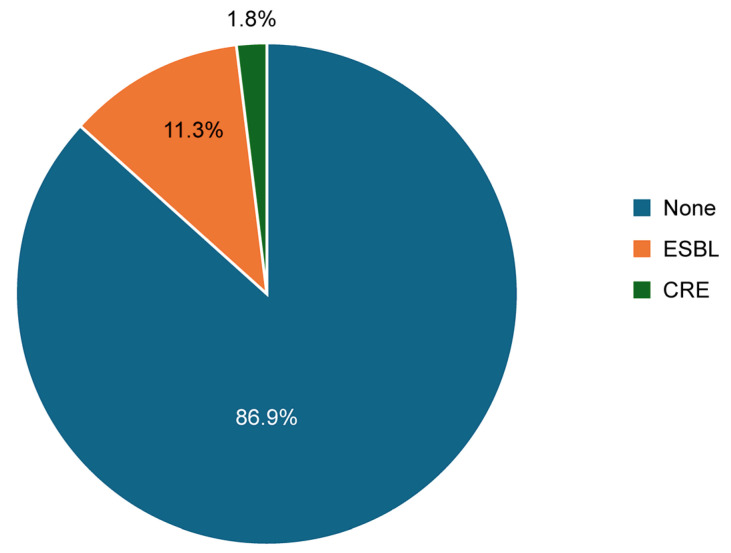
Rate of ESBL-producing Enterobacterales and CRE among the studied population.

**Figure 2:**
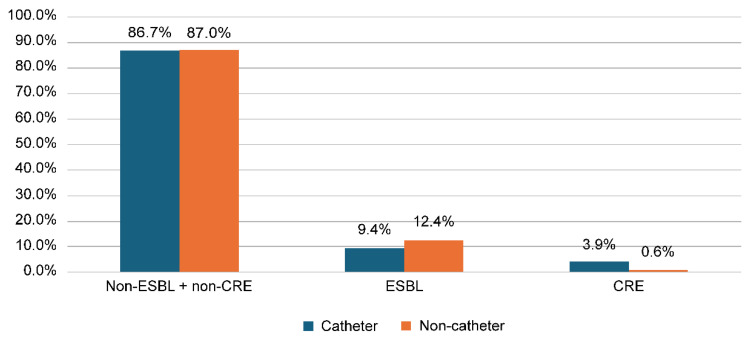
Distribution of ESBL and CRE according to catheterization status among the studied population.

Figure 1 shows that, out of the total urine samples analyzed, the prevalence of ESBL-producing Enterobacterales was 11.3%, while CRE accounted for 1.8%. As shown in Figure 2, ESBL infections were more prevalent among non-catheterized patients (12.4%) compared to catheterized patients (9.4%). In contrast, CRE infections were more common in catheterized patients (3.9%) than in non-catheterized patients (0.6%).

### 3.2. Predominant Bacterial Species and Distribution and Molecular Resistance Patterns

**Figure 3:**
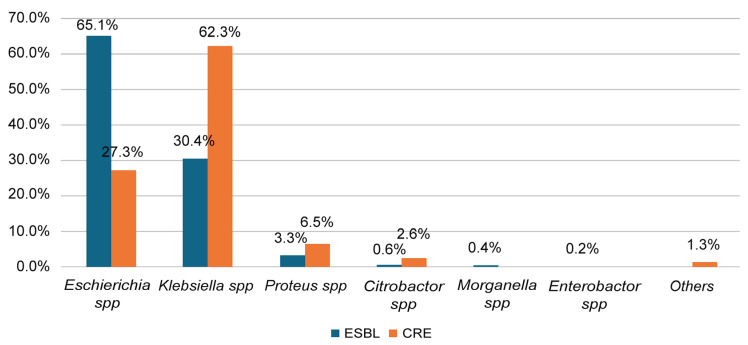
Prevalence of ESBL and CRE according to bacterial species in the present study.

Figure 3 depicts that among the ESBL isolates, *E. coli* spp. exhibited the highest prevalence (65.1%), followed by *Klebsiella* spp. (30.4%). In contrast, *Klebsiella* spp. was the most prevalent CRE organism (62.3%), followed by *E. coli* spp. (27.3%). Additionally, in both ESBL and CRE groups, *Proteus*, *Citrobacter*, and *Morganella* spp. were the least frequently detected organisms.

This study identified a variety of CRE organisms isolated from urine samples, with some species showing higher prevalence and stronger associations with specific resistance genes. *Klebsiella* spp. was the most detected organism among all CRE isolates, followed by *E. coli*. Additional species, including *Proteus*, *Citrobacter*, *Morganella*, and a group of less common organisms categorized as “Others,” were also found. Molecular analysis showed that all CRE isolates in this study carried either the *bla-OXA-48* gene, the *bla-NDM* gene, or both, with no additional carbapenemase genes detected.

**Figure 4:**
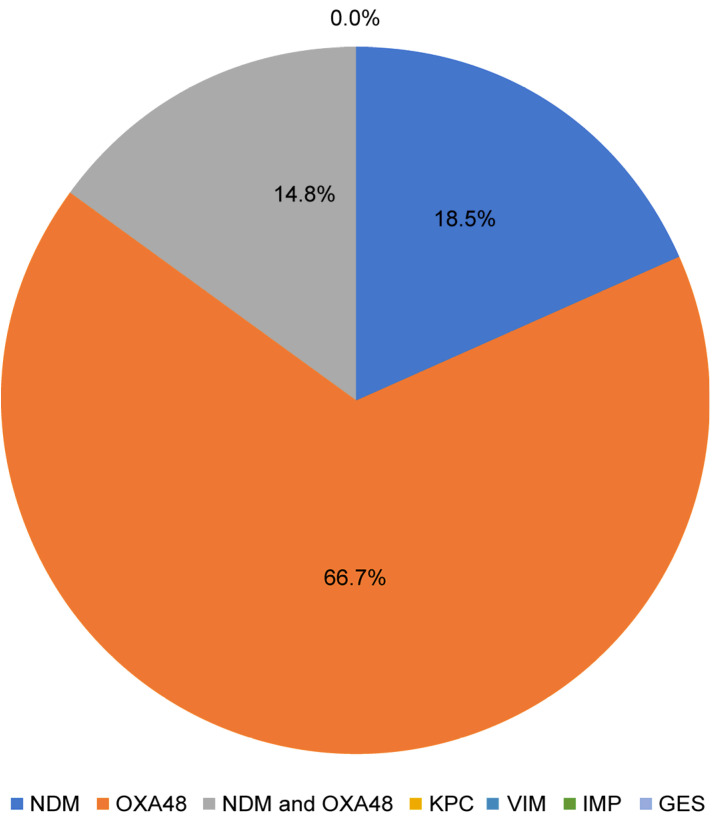
Distribution of Genes Amongst CRE in our cohort.

*Bla-OXA-48* was the most prevalent carbapenemase gene, found in 66.7% of CRE isolates, as illustrated in Figure 4. A total of 18.5% of isolates had the *bla-NDM* gene, while 14.8% harbored both genes simultaneously. This distribution highlights *bla-OXA-48* as the predominant resistance mechanism among CRE in our setting, with a considerable portion of isolates exhibiting dual gene carriage.

Supplementary Figure S1 shows the distribution of these genes among bacterial species. *Klebsiella* spp. represented the majority of gene-positive isolates, accounting for 46.7% of all *bla-NDM*-positive, 68.5% of *bla-OXA-48*-positive, and 66.7% of dual-gene isolates. *E. coli* was the second most common species, contributing 26.7% of *bla-NDM*-positive, 13% of *bla-OXA-48*-positive, and 8.3% of dual-gene isolates. *Proteus* spp. accounted for 10.7% of *bla-NDM*, 9.3% of *bla-OXA-48*, and 5.4% of dual-gene detections. *Citrobacter* spp. contributed 3.0% of *bla-NDM*, 1.9% of *bla-OXA-48*, and 8.3% of dual-gene isolates. *Morganella* spp. represented 3.0%, 1.9%, and 5.4% of *bla-NDM*, *bla-OXA-48*, and dual-gene cases, respectively. “Other” species comprised 9.9% of *bla-NDM*, 5.4% of *bla-OXA-48*, and 5.9% of dual-gene detections.

### 3.3. Patient Characteristics and Potential Risk Factors for ESBL and CRE Infections

In the sample of both ESBL- and CRE-positive patients (Supplementary Table S2), ESBL was more frequently detected in non-catheterized specimens (68.8%) and emergency settings (67.8%), whereas CRE was predominantly associated with catheterized samples (80.5%) and inpatient settings (72.7%). Supplementary Table S2 further shows that CRE infections were more common in older patients (>45 years, 81.8%), males (50.6%), and those with urinary catheters (80.5%). In contrast, ESBL infections were more prevalent in younger patients (≤45 years, 56.1%) and females (77.3%). Additionally, CRE cases were primarily identified in inpatient settings (72.7%), while ESBL cases were more frequent in emergency settings (67.8%). Further subgroup analysis of ESBL and CRE (Table 1) by specimen type revealed distinct demographic and clinical profiles.

Further analysis of ESBL cases (Table 1) showed that catheter-associated infections were significantly more likely to occur in inpatients (29.3% vs. 6.0%, *p* < 0.001). Conversely, non-catheter-associated ESBL cases were most prevalent in emergency (69.2%) and outpatient (24.8%) settings, highlighting a clear distinction in clinical distribution based on catheter use.

As shown in Table 1, no statistically significant associations were identified within the CRE group across demographic or clinical variables. Specifically, no significant relationships were observed between specimen type and patient age, sex, nationality, or care setting (all *p* > 0.05).

Supplementary Figure S2 compares ESBL and CRE prevalence according to catheterization status across all urine samples tested, rather than limiting the analysis to positive subsets alone, as done in Table 1. The results show that ESBL prevalence was higher among non-catheterized patients (12.4%) compared to catheterized patients (9.4%), whereas CRE prevalence was higher among catheterized patients (3.9%) compared to non-catheterized patients (0.6%). Additionally, *E. coli* and *Klebsiella* spp. were the most frequently identified microbial species among ESBL and CRE groups, respectively. ESBL infections were most frequently observed in the outpatient department (67.4%), while CRE infections were predominantly reported in OPD (44.2%) and inpatient surgical wards (23.4%).

## 4. Discussion

### 4.1. Prevalence of ESBL and CRE in Enterobacteriales Isolates and the Impact of Catheterization

Results showed a significantly higher rate of ESBL-producing organisms compared to CRE among all positive Enterobacterales. ESBL was more frequently identified in non-catheterized patients and in emergency care settings, whereas CRE was mainly linked to catheterized inpatients. These findings align with previous reports demonstrating a community-associated increase in ESBL [8,9], and nosocomial acquisition of CRE infections [16,17].

Notably, ESBL was associated with younger age groups and female patients, whereas CRE infections were more frequent in older men. This suggests differing epidemiological profiles and transmission pathways for the two resistance mechanisms. The prevalence of ESBL in emergency settings may reflect empirical antibiotic use outside outpatient care, limited post-treatment follow-up, and/or pre-treatment antibiotic exposure. This is further supported by the higher incidence among female patients, which underscores the community-acquired nature of ESBL, as women are more likely to develop UTIs. In contrast, CRE infections were more common in older men in the inpatient setting, where catheter use is prevalent, highlighting the correlation between CRE and catheterization.

The absence of major associations for CRE infections with demographic variables further suggests that clinical status, underlying comorbidities, or prior healthcare exposure may play a more substantial role—factors not fully captured in this retrospective dataset.

### 4.2. Predominant Bacterial Species and Molecular Resistance Patterns

All CRE isolates were positive for *bla-OXA-48*, *bla-NDM*, or both in our molecular data. *bla-OXA-48* was the most frequent, followed by *bla-NDM* and co-expression of both genes. This finding is consistent with previous studies from various regions in Saudi Arabia, which reported *bla-OXA-48* as the most prevalent gene among isolates with carbapenem resistance [18,19,20].

In contrast, data from other parts of the world show a wider diversity of resistance genes and identify *bla-KPC* as the most prevalent gene in regions such as North America, Europe, and parts of Asia [21].

*Klebsiella* spp. were the main carriers of carbapenemase genes, especially with dual gene isolates, while *E. coli* and *Proteus* spp. had a greater majority of *bla-NDM* genes. This is consistent with global surveillance data and Saudi Arabia-based data, indicating the importance of *Klebsiella* as a CRE reservoir [19,21]. Moreover, although *E. coli* has a minor role in the carriage of CRE genes and their dissemination, *bla-NDM* genes are still of significant importance as they are transmitted via plasmid, which contributes to resistance spread.

*Klebsiella pneumoniae* is a major driver of antimicrobial resistance globally, serving as a key reservoir and disseminator of carbapenemase genes, including *bla-OXA-48* and *bla-NDM*. Beyond intrinsic resistance mechanisms, *Klebsiella* possesses several virulence factors, notably siderophores, which enhance its pathogenicity and contribute to hypervirulent phenotypes. Understanding these epidemiological and pathogenic features is essential to contextualize the role of this species in the local molecular resistance landscape [22,23,24].

Our findings highlight the ongoing challenges of antimicrobial resistance in Saudi Arabia and provide local data that may contribute to regional surveillance attempts. While *bla-OXA-48* and *bla-NDM* predominate locally, global trends, such as the rise of *bla-KPC* and other gene families, underscore the importance of broader gene panel screening in diagnostic laboratories. Strengthening surveillance networks across the region will be essential to track these shifts and prevent the establishment of endemic resistance patterns.

### 4.3. Patient Characteristics and Potential Risk Factors for ESBL and CRE Infections

Our demographic data show that overall cases were more common in females, likely due to anatomical susceptibility to UTIs. Catheterized patients and those older than 45 years were more likely to harbor CRE, further supporting the association of risk factors with invasive devices and advanced age. These trends are consistent with findings by Natoubi et al., Zhang et al., and Lin et al., who identified extended hospitalization, catheterization, and comorbidities such as diabetes and renal insufficiency as major contributors to resistance [14,17,25].

The relatively high proportion of ESBL in non-catheterized patients, particularly in emergency settings, indicates that community-acquired resistance is becoming more prominent. These findings align with earlier Saudi-based research by Abalkhail et al. and underscore the need for community-focused antibiotic stewardship interventions [10].

Although only a few of our cases were admitted to the ICU, underreporting or underrepresentation may have contributed to this finding. However, previous administration of broad-spectrum antibiotics, mechanical ventilation, or the existence of critical illness in an ICU have been established as risk factors for CRE emergence [4].

### 4.4. Summary of Findings: Clinical Implications

These findings underscore the need for aggressive infection control among hospitalized, catheterized patients to prevent CRE transmission. Additionally, the increasing resistance in outpatients and emergency settings calls for an urgent reassessment of empirical antibiotic regimens, especially in light of the frequent overuse of third-generation cephalosporins.

The dissemination of *bla-OXA-48* and *bla-NDM* genes highlights the need for continuous molecular monitoring to track resistance trends and guide appropriate treatment. This should be coupled with improved catheter hygiene, timely removal protocols, and robust antimicrobial stewardship programs.

Owing to the global escalation of antimicrobial resistance, there is a critical need to explore novel therapeutic strategies beyond conventional antibiotics. Recent approaches have focused on exploiting bacterial iron acquisition systems through siderophore–drug conjugates. This strategy, known as the “Trojan horse” strategy, enables antibiotics to bypass resistance mechanisms by hijacking bacterial nutrient transporters. Additionally, targeting bacterial metallophores represents a promising avenue for the development of entirely new classes of antimicrobials [26,27,28].

Sex- and age-based patterns identified in this study may enable risk stratification in clinical practice. For example, older male inpatients with urinary catheters should be considered high risk for CRE and managed accordingly.

### 4.5. Application

The results of this study align with recent Infectious Diseases Society of America guidelines recommending a reduction in unnecessary and prolonged use of indwelling urinary catheters to prevent catheter-associated infections, including those caused by ESBL and CRE. Additionally, the widely endorsed “3-day rule,” which suggests reassessing catheter necessity within 72 h, supports our findings, particularly given the significantly higher CRE prevalence among catheterized patients. Implementing such best practices can reduce biofilm formation and bacterial colonization, especially in high-risk hospital wards, and represents a critical step toward limiting the spread of resistant Enterobacterales in both inpatient and long-term care settings [25,28].

### 4.6. Limitations and Recommendations

Future research should consider long-term trends in antibiotic susceptibility among urinary Enterobacterales isolates. Longitudinal studies would be particularly useful in addressing this limitation, supporting more effective antibiotic stewardship, and refining clinical guidelines. The absence of resistance trend data and the inability to track patient status throughout hospitalization are notable limitations. Moreover, as this was a retrospective, single-center study conducted over 2 years, the findings may not be generalizable to broader populations or healthcare settings. By nature, these limitations constrain the ability to establish causality and conduct a robust analysis of long-term resistance trends.

## 5. Conclusions

This study provides insights into ESBL-producing Enterobacterales and CRE in urinary infections across over 4000 patient samples. The present study illustrates the presence of high resistance rates among urinary Enterobacterales isolates, particularly among catheterized patients, hospitalized individuals, older adults, and men. ESBLs were more frequently found in women and outpatient settings, indicating growing resistance within the community. The high prevalence of *Klebsiella* spp., *E. coli*, and resistance genes, such as *bla-OXA-48* and *bla-NDM*, emphasizes the importance of conducting continuous molecular surveillance in this population. These findings underscore the urgent need for enhanced infection control, judicious antibiotic use, and individualized treatment strategies to improve patient outcomes and curb the spread of resistant infections.

## Figures and Tables

**Table 1:** Relationship between specimen type according to demographic characteristics in patients with ESBL (*n* = 418) and patients with CRE (*n* = 77).

Factor	*ESBL* *Specimen Type*		*p*-Value §	*CRE* *Specimen Type*		*p*-Value §
	*Catheter* *N (%)* *(* *n = 150)*	*Non-Catheter* *N (%)* *(n = 331)*		*Catheter* *N (%)* *(* *n = 62)*	*Non-Catheter* *N (%)* *(* *n = 15)*	
Age group						
≤45 years	76 (50.7%)	194 (58.6%)	0.104	13 (21.0%)	01 (6.7%)	0.280
>45 years	74 (49.3%)	137 (41.4%)		49 (79.0%)	14 (93.3%)	
Sex						
Male	41 (27.3%)	68 (20.5%)	0.099	34 (54.8%)	05 (33.3%)	0.160
Female	109 (72.7%)	263 (79.5%)		28 (45.2%)	10 (66.7%)	
Nationality						
Saudi	129 (86.0%)	281 (84.9%)	0.751	48 (77.4%)	14 (93.3%)	0.277
Non-Saudi	21 (14.0%)	50 (15.1%)		14 (22.6%)	01 (6.7%)	
Type						
Emergency	97 (64.7%)	229 (69.2%)	<0.001 **	13 (21.0%)	04 (26.7%)	0.227
Inpatient	44 (29.3%)	20 (6.0%)		47 (75.8%)	09 (60.0%)	
Outpatient	09 (6.0%)	82 (24.8%)		02 (3.2%)	02 (13.3%)	

§ *p*-values are calculated using the Chi-square test. ** Significant at *p* < 0.05.

## Data Availability

The data presented in this study are available on request from the corresponding authors due to privacy and ethical considerations.

## Supplementary Materials

The following supporting information can be downloaded at https://www.mdpi.com/article/10.3390/medicina61111907/s1: Figure S1: Distribution of genes according to bacterial species; Figure S2: Prevalence of ESBL and CRE according to ward; Table S1: Demographic characteristics of the study population (N = 4262); Table S2: Relationship between ESBL and CRE according to patient demographic and clinical characteristics (N = 558).
