# Supplementary material for: Urinary Tract Infections Caused by Extended-Spectrum Beta-Lactamase-Producing and Carbapenem-Resistant Enterobacterales in Saudi Arabia: Impact of Catheterization
Source: Medicina (Kaunas). 2025 Oct 24;61(11):1907. doi: 10.3390/medicina61111907 (PMC12654839; doi:10.3390/medicina61111907)
Supplement: Supplementary file 1 [file medicina-61-01907-s001.zip › medicina-3886712-supplementary.pdf]

Supplementary Materials

Supplementary Table S1. Demographic characteristics of the study population (N=4262)

Study variables	N (%)
Age group	
· 0–17 years	628 (14.7%)
· 18–30 years	637 (14.9%)
· 31–40 years	647 (15.2%)
· 41–50 years	515 (12.1%)
· 51–60 years	487 (11.4%)
· 61–70 years	550 (12.9%)
· >70 years	798 (18.7%)
Sex	
· Male	1322 (31.0%)
· Female	2940 (69.0%)
Nationality	
· Saudi	3542 (83.1%)
· Non-Saudi	720 (16.9%)
Area name	
· Outpatient: Emergency	2380 (55.8%)
· Outpatient: OPD	904 (21.2%)
· Inpatient: Medical Ward	510 (12.0%)
· Inpatient Surgical ward	292 (6.9%)
· Inpatient: OB-gynae	78 (1.8%)
· Inpatient: Pediatric ward	53 (1.2%)
· Inpatient: Medical ICU/NICU	45 (1.0%)

Abbreviations: OPD, outpatient department; ICU, intensive care unit; NICU, neonatal intensive care unit.

Supplementary Table S2. Relationship between ESBL and CRE according to patient demographic and clinical characteristics (N=558)

Factor	ESBL N (%) (n=481)	CRE N (%) (n=77)	P-value §
Age group			
· ≤45 years	270 (56.1%)	14 (18.2%)	<0.001 **
· >45 years	211 (43.9%)	63 (81.8%)	
Sex			
· Male	109 (22.7%)	39 (50.6%)	<0.001 **
· Female	372 (77.3%)	38 (49.4%)	
Nationality			
· Saudi	410 (85.2%)	410 (85.2%)	0.287
· Non-Saudi	71 (14.8%)	15 (19.5%)	
Specimen type			
· Catheter	150 (31.2%)	62 (80.5%)	<0.001 **
· Non-catheter	331 (68.8%)	15 (19.5%)	
Type			
· Emergency	326 (67.8%)	17 (22.1%)	<0.001 **
· Inpatient	64 (13.3%)	56 (72.7%)	
· Outpatient	91 (18.9%)	04 (5.2%)	

§ P-value has been calculated using Chi-square test
** Significant at P<0.05.

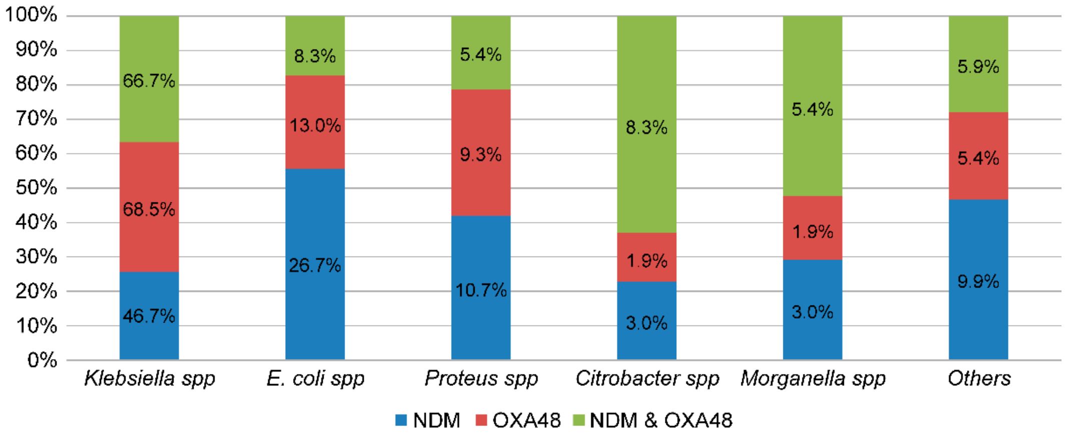

Figure S1. Distribution of genes according to bacterial species

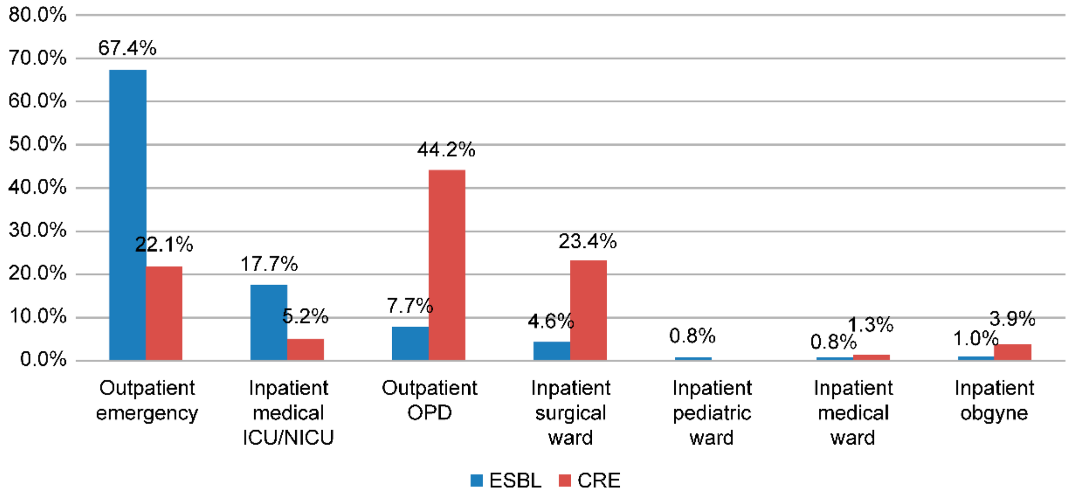

Figure S2. Prevalence of ESBL and CRE according to ward
